# Close of the College Session

**Published:** 1867-03

**Authors:** 


### CLOSE OF THE COLLEGE SESSION.

The Commencement exercises of the Ohio College of Dental Surgery were held in the amphitheater of the College on Wednesday evening, March 6th. The degree of "Doctor of Dental Surgery" was conferred, by James Taylor, M. D., D. D. S., President of the Board of Trustees, on the following members of the Senior Class:

| | | |
|---|---|---|
| W. C. Stanley, | H. L. Ambler, | W. A. Grahame, |
| Frank McGinnis, | J. T. Child, | R. F. Ludwig, |
| F. Peabody, | Byron Eaton, | J. R. Irelan, |
| G. W. Field, | P. T. Clark, | J. Ropp. |

He also, in the name of the College, presented each of them with a handsome copy of the Holy Bible, this being a parting gift of the institution to all its alumni. His address to the candidates was heard with interest, not only by them, but by the large audience assembled.

The valedictory on behalf of the faculty, was by Dr. Joseph Richardson, late a professor in the college, but was read by Prof. Spalding, Dr. R. having been detained by the death of a friend. The response from the class was read by Dr. W. A. Grahame. The proceedings were brief, appropriate, and well received. The Dean of the faculty announced that the session of 1866–'67 was now closed, having lasted three weeks longer than any previous one; and that the next session would begin next October, and continue five, and possibly, six months. The audience was dismissed with the "benediction," by ——— ———, who had appropriately opened the meeting by prayer, and reading and paraphrasing a portion of the sacred scriptures. The exercises were attended by many members of the profession from abroad, who have the thanks of the faculty for their interest in our labors. The names of those who received honorary degrees will be published in a future number. W.

